# How effective are mood stabilizers in treating bipolar patients comorbid with cPTSD? Results from an observational study

**DOI:** 10.1186/s40345-024-00330-1

**Published:** 2024-03-26

**Authors:** Anna Maria Iazzolino, Marta Valenza, Martina D’Angelo, Grazia Longobardi, Valeria Di Stefano, Steardo Luca, Caterina Scuderi, Luca Steardo jr

**Affiliations:** 1 Psychiatry Unit, Department of Health Sciences, University of Catanzaro Magna Graecia, Catanzaro, 88100 Italy; 2 Department of Physiology and Pharmacology “Vittorio Erspamer”, SAPIENZA University of Rome, Rome, Italy; 3 Università Giustino Fortunato, Benevento, 82100 Italy

**Keywords:** Bipolar disorder, Complex posttraumatic stress disorder, Clinical correlates, Lithium, Treatment response, Alda Scale

## Abstract

**Background:**

Multiple traumatic experiences, particularly in childhood, may predict and be a risk factor for the development of complex post-traumatic stress disorder (cPTSD). Unfortunately, individuals with bipolar disorder (BP) are more likely to have suffered traumatic events than the general population. Consequently, cPTSD could be comorbid with BD, and this may negatively affect psychopathological manifestations. To date, no one has explored whether such comorbidity also affects the response to treatment with mood stabilizers in BD patients.

**Results:**

Here, a cross-sectional study was carried out by comparing the response to treatment, measured by the Alda scale, in a cohort of 344 patients diagnosed with BD type I and II, screened for the presence (or absence) of cPTSD using the International Trauma Questionnaire. The main result that emerged from the present study is the poorer response to mood stabilizers in BD patients with comorbid cPTSD compared with BD patients without cPTSD.

**Conclusions:**

The results collected suggest the need for an add-on therapy focused on trauma in BD patients. This could represent an area of future interest in clinical research, capable of leading to more precise and quicker diagnoses as well as suggesting better tailored and more effective treatments.

## Introduction

In 2018, the 11th Revision of the International Classification of Diseases (ICD-11) formally acknowledged Complex Post-Traumatic Stress Disorder (cPTSD) as an independent syndrome distinct from PTSD. Both are classified under the main category of “Disorders specifically associated with stress” which comprises severe mental disorders that develop following exposure to an event or series of events of an extremely threatening or horrific nature, from which escape is difficult or impossible (e.g., torture, slavery, genocide campaigns, prolonged domestic violence, repeated childhood sexual or physical abuse) (ICD-11 for Mortality and Morbidity Statistics). In addition to the three main symptoms of PTSD (reexperience, avoidance, and hypervigilance), cPTSD symptoms include severe and perduring impairments in three domains of self-organization (DSO) which are emotional dysregulation, negative self-concept, and interpersonal difficulties (Nestgaard Rød and Schmidt 2021). The prevalence of the disorder in the general population varies between 1 and 8%, while in psychiatric institutions it reaches up to 50% (Maercker et al. 2022), which increases the probability of developing other psychiatric disorders such as anxiety disorders and major depression (Karatzias et al. 2019a; Letica-Crepulja et al. 2020; Murphy et al. 2021), bipolar disorder (BD) and personality disorders (Lewis et al. 2022) and increases the risk of suicidality (Karatzias et al. 2019a). When different mental illnesses co-occur, it is likely that there are common risk factors contributing to the onset of these conditions. In the case of comorbid cPTSD with BD, the common factor is trauma. It is known that individuals with BD are more likely than the general population to have experienced traumatic events. Estimates suggest that there is a high rate of PTSD in BD ranging from 6 to 55% (Cerimele et al. 2017) even if there is currently little data on cPTSD prevalence specifically in BD. Although childhood adversity may predict and serve as a risk factor for the development of cPTSD, as demonstrated by Maercker and colleagues, there are high rates in general psychiatric settings of cPTSD (Maercker et al. 2022; Tian et al. 2022). Cumulative trauma, defined as “the consequences of experiencing various and multiple types of traumas over time,” can lead to complex PTSD and result in more severe symptoms of psychopathology compared to single-event traumas. This issue is common in bipolar disorder and has a negative impact on its course, such as rapid cycling, a higher number of episodes, and psychotic symptoms (Kira 2010; Shevlin et al. 2008), but little is known about its effect on treatment response. Studies in the literature have primarily focused on the impact of childhood trauma and considered it a potentially important factor in the treatment of mood disorders (Agnew-Blais and Danese 2016; Cotter et al. 2015). Several studies have shown an association between childhood trauma and poorer response to pharmacotherapy in adolescents and adults diagnosed with major depressive disorder (Klein et al. 2009; Shamseddeen et al. 2011; Douglas and Porter 2012; Nanni et al. 2012). However, there are only a few studies that have examined a similar association in BD (Etain et al. 2017; Cakir et al. 2016; Cascino et al. 2021; Benarous et al. 2017; Perugi et al. 2018) although some reports suggest that childhood trauma may attenuate treatment outcomes in this population. In 2017 Etain and collaborators showed that higher levels of physical abuse were associated with poorer response to lithium measured with the Alda Scale (Scott et al. 2020). Also, patients who had experienced multiple types of childhood trauma (i.e., physical, sexual, or emotional abuse) were more likely to have an inadequate response to mood stabilizers than patients who had not been exposed to traumatic events in childhood (Etain et al. 2017). Furthermore, a recent meta-analysis conducted by Wrobel and colleagues have focused on how childhood trauma may influence the response to pharmacological treatment in adolescents with BD (Wrobel et al. 2022).

Cascino and colleagues (2021) confirmed in a small sample of adult BD patients that a history of childhood abuse influences clinical response to lithium, but not to other mood stabilizers. In contrast, comorbid PTSD seems to affect the response to treatment of both quetiapine and lithium in patients with BD (Russell et al. 2023).

This suggests that comorbid PTSD has an impact on the therapeutic management of BD. However, to the best of our knowledge, no study has yet examined the impact of cPTSD on response to mood stabilizer treatment in adult patients affected by BD. Therefore, the present paper aims to contribute to filling this gap by studying the response to pharmacological treatment in BD patients with and without cPTSD. To do so we used the Alda scale, an 11-point rating scale (Grof et al. 2002) adapted to measure the response to mood stabilizers (Garnham et al. 2007; Steardo et al. 2019). Based on the aforementioned literature, we hypothesized that BD patients with comorbid cPTSD would respond less well to treatment than patients who had never been exposed to trauma.

## Methods

### Study design, recruitment of participants, and eligibility criteria

The study here presented is observational and cross-sectional, and the methods used are reported according to the STROBE checklist (https://www.equator-network.org/reporting-guidelines/strobe/). Patients were consecutively enrolled by the Unit of Psychiatry of the University of Catanzaro “Magna Graecia”. Three hundred and forty-four patients were included in the study and followed up as outpatients from February 2021 to August 2023. Each participant was adequately informed about the purpose of the research protocol, the protection of personal data, and the preservation of privacy and anonymity. Participation in the study was voluntary and required the patient to sign a formal consent form.

### Inclusion and exclusion criteria

The inclusion criteria were the following: (1) having been diagnosed with BD according to criteria of the Diagnostic and Statistical Manual of Mental Disorders (DSM-5), assessed by clinical interviews and administration of psychometric scales (SCID-5-CV); (2) age between 18 and 75 years; (3) written informed consent to participate in the study; (4) stable adequate treatments with mood stabilizers (at least 1-year duration and, in the case of lithium, at therapeutic blood levels).

Exclusion criteria were the following: (1) inability to provide written consent; (2) diagnosis of major neurological and psychiatric diseases (such as epilepsy, cognitive disabilities, and genetic syndromes with psychiatric manifestations); (3) the presence of any condition that did not allow to complete the overall assessment, such as language barriers and severe specific cognitive disabilities (e.g. dyslexia).

After recruitment, participants underwent a series of clinical and psychopathological assessments during outpatient clinical visits. Each enrolled patient underwent a semi-structured clinical interview in order to gather clinical and medical history.

Patients clinical and sociodemographic characteristics, including gender, age at study entry, employment status, educational level, family history of psychiatric illnesses, type of onset, lifetime number of affective episodes, the pattern of illness course, treatments, suicidal ideation, and previous psychiatric hospitalizations, the presence of trauma were recorded according to an ad-*hoc* schedule. In the schedule we developed for this study, there were a series of questions aimed at investigating the presence of trauma, specifically: natural disasters; serious work-related accidents or potentially life-threatening injuries (confirmed through medical reports); physical abuse; sexual abuse; and a subsection labeled “other,” where we described the particularly stressful event witnessed. Therefore, each participant underwent two psychometric assessments, the International Trauma Questionnaire (ITQ) and the Alda Scale.

### Psychometric tools

The ITQ was used to assess the trauma experienced and to diagnose cPTSD according to the ICD-11 guidelines. The questionnaire consists of eighteen items measuring the main symptoms of PTSD and DSO (Cloitre et al. 2018, 2021; Rossi et al. 2022). The items can be answered on a 5-point Likert scale from 0 (not at all) to 4 (very strongly). The maximum score for PTSD and/or DSO is therefore 24 (range 0–24), while the maximum score for cPTSD is 48 (range 0 to 48). All items can only be considered present if they have a value ≥ 2 on the Likert scale. Diagnosis of cPTSD requires the endorsement of one of two symptoms from each of the three PTSD symptom clusters (re-experiencing, avoidance, and sense of current threat) and one of two symptoms from each of the three Disturbances in Self-Organization (DSO) clusters: (1) affective dysregulation, (2) negative self-concept, and (3) disturbances in relationships. Functional impairment is present when at least one cluster of functional impairment is associated with PTSD symptoms and one with DSO symptoms. In principle, a person can only receive one of the two diagnoses, namely PTSD or cPTSD. Furthermore, cPTSD is characterized by the predominance of symptoms of disturbances in self-organization and is defined as that set of symptoms resulting from cumulative interpersonal traumas experienced during development: stories of abuse and repeated maltreatment in the family, severe neglect and abandonment, conditions of torture or imprisonment, wars, and forced migrations. When a person cannot escape the threat for a long time or when the threat occurs within the family from which one must continue to depend for survival, the mind deploys more intense strategies to overcome the paradox and the pervasive state of fear: this is referred to as chronic traumatization rather than a single traumatic event.

Response to mood stabilizer treatment was assessed using the mean score of the retrospective criteria for long-term treatment response in BD (Alda Scale), which consists of two criteria: (A) rating of the association between clinical improvement and treatment, and (B) rating of the degree of causal relationship between clinical improvement and prophylactic treatment. The total score was determined by subtracting the B score from the A score. Subjects were scored on a 0 − 10 scale. A total score of ≥ 7 indicates a good response, a score from 4 to 6 a moderate response, and a score of ≤ 3 a poor response. The Alda scale is a valid measure with interrater reliability of 0.54–0.75 in assessing long-term response to treatment (Manchia et al. 2013; Steardo et al. 2019). The study was carried out in accordance with the latest version of the Declaration of Helsinki and was approved by the Ethics Committee of the Calabria Region (N. 307/2020).

### Statistical analysis

Statistical analysis was carried out using the Statistical Package for Social Sciences Version 26 (SPSS, Chicago, Illinois, USA). Descriptive analyses were carried out to evaluate the distribution of the variables in the whole sample and the normality distribution was assessed through Shapiro-Wilk’s test. Subjects were divided into two groups according to the presence/absence of cPTSD. Groups were compared using the Chi-square or the Student’s *t*-test depending on the type of variable. The logistic regression model was used to analyze the association between the presence of cPTSD and the response to treatment with mood stabilizers. The level of statistical significance was set at a value of *p* ≤ 0.05.

## Results

### Sociodemographic and clinical description of the sample

Three hundred and forty-four patients met the inclusion criteria and were included in the study. The demographic information shown in Table 1 provides insights into the gender distribution, educational background, marital status, and employment status of the sample. The prevalence of certain clinical characteristics including age of onset of BD, family history, aggressive behaviors, and psychiatric symptoms further contributes to the overall profile of this patient cohort. A schematic description of all such features is reported in Table 1. In detail, the most frequent diagnosis was BD of type I (*N* = 253, 73.5%), compared with BD of type II (*N* = 91, 26.5%). The mean age was 47 years old (± 14), and the age at onset of BD was about 26 years (± 9.4). The patients were rather evenly distributed by sex (Female *N* = 177, 51.5%; Male *N* = 167, 48.5%) and marital status (*N* = 186, 54.1%). A significant majority of them completed their education (71.5%), but only half of the cohort were employed (*N* = 171, 49.7%).

The duration of untreated illness was for approximately 6 years (± 10.3), and the average number of affective episodes was 11.4 (± 9.87). This included an average of 5.81 (± 5.92) depressive episodes, 4.04 (± 4.41) manic episodes, and 2.65 (± 2.69) hypomanic episodes. Nearly half of the individuals (49.7%) reported seasonality features. More than half of the cohort of patients presented aggressive behaviors (*N* = 194, 56.4%) and mixed features (*N* = 193, 56.1%). Approximately 42% reported psychotic symptoms, whereas 24.7% experienced mania triggered by antidepressant use. Suicide attempts were reported by 40.1% of enrolled patients.

Table 2 shows the types of treatments in the whole sample. Of the 344 patients enrolled in the study 67 were on lithium treatment (mean daily dose = 900 ± 240 mg/day), 55 (19.5%) on lithium with valproic acid and quetiapine, 47 (13.6%) on a combination of quetiapine with either valproate (*N* = 47, 13.6%). The other most used stabilizer was lamotrigine, either in combination with lithium or alone with an antipsychotic.

**Table 1:** Description of the cohort of BD patients enrolled in the study (*N* = 344) and results from sociodemographic and clinical assessment

Characteristic	M or N	± SD or %
Age, M (± SD)	46.88	± 13.94
Age at onset, M (± SD)	25.73	± 9.40
duration of untreated illness, M (± SD)	5.84	± 10.31
number of depressive episodes, M (SD±)	5.81	± 5.92
number of manic episodes, M (± SD)	4.04	± 4.41
number of hypomanic episodes, M (SD±)	2.65	± 2.69
numbers of total affective episodes, M (± SD)	11.39	± 9.87
Female, N (yes%)	177	51.5%
Graduation, N (yes%)	246	71.5%
Marital status, N (yes%)	186	54.1%
Employed, N (yes%)	171	49.7%
Diagnosis of bipolar disorder I, N (yes%)	253	73.5%
Diagnosis of bipolar disorder II, N (yes%)	91	26.5%
Family history of psychiatric disorder, N (yes%)	190	55.2%
Seasonality, N (yes%)	171	49.7%
Aggressive behaviors, N (yes%)	194	56.4%
Mixed features, N (yes%)	193	56.1%
Lifetime abuse, N (yes%)	124	36.0%
Psychotic symptoms, N (yes%)	144	41.9%
Antidepressant mania, N (yes%)	85	24.7%
Suicide attempts, N (yes%)	138	40.1%
cPTSD Prevalence, N (yes%)	154	44.7%

Note. Mean (M), Numerosity (N), standard deviation (SD)

**Table 2:** Description of the therapeutic management of the BD patients enrolled in the study (*N* = 344)

	N	%
Li	67	19.4
Li + qutiapine	17	4.9
Li + olanzapine	10	2.9
Li + lurasidone	5	1.4
Li + dvp + quetiapine	55	15.9
Li + dvp + olanzapine	27	7.8
Li + lamotrigine + quetiapine	26	7.5
Li + lamotrigine + olanzapine	24	6.9
Li + dvp + aripiprazole	9	2.6
dvp + qutiapine	47	13.6
dvp + olanzpine	22	6.3
dvp + lurasidone	4	1.1
dvp + aripiprazole	9	2.6
lamotrigine + olanzapine	9	2.6
lamotrigine + quetiapine	7	2.03
carbamazepine + quetiapine	6	1.7

Note. lithium (Li); valproic acid (dvp)

### Identification of BD patients with comorbid cPTSD using the ITQ score

The ITQ questionnaire was administered to all BD patients recruited in the study (*N* = 344) and the analysis of the distribution of our sample in both PTSD and DSO domains is displayed in Table 3.

For each PTSD symptom cluster (Re-experiencing, Avoidance, and Hyperarousal), the mean scores are relatively low, indicating a generally lower level of severity in these specific symptoms.

Within the DSO symptom cluster, both Affective Dysregulation and Disturbances in Relationships show slightly higher mean scores, suggesting a comparatively higher level of impairment in these areas. Instead, the Negative Self-Concept cluster shows a lower mean but a wider interquartile range (IQR), indicating greater variability in the severity of this symptom among individuals in the sample. Overall, these results provide insights into the distribution and severity of PTSD and DSO symptoms within the studied population, helping to understand the impact of trauma on different aspects of their mental health.

**Table 3:** Results of the administration of the ITQ to the whole sample (*N* = 344), describing the distribution of patients in each symptom cluster

		Mean	SD	Median	IQR
PTSD scores	**Re-experiencing**	1.48	1.42	1	3
	**Avoidance**	1.29	1.34	1	3
	**Hyperarousal**	1.46	1.34	1	3
DSO scores	**Affective dysregulation**	1.72	1.66	1	3
	**Negative self-concept**	1.32	1.45	1	4
	**Disturbances in relationships**	1.69	1.68	1	4

DSO, *Disturbances in Self-Organization*; PTSD, *Post-traumatic stress disorder*.

Considering the ITQ score, 44.8% of enrolled patients (*N* = 154) met the criteria for cPTSD diagnosis, and their characteristics are summarized in Table 4. By comparing the sociodemographic and clinical characteristics of patients between the two groups, we detected statistical differences in gender distribution and educational background between subjects who met the criteria for cPTSD and those who did not. Marital status shows a significant difference, with a higher percentage of individuals being married in the cPTSD group whereas employment status does not show a significant difference between the two groups.

The group with cPTSD tended to be relatively younger, even at the onset of BD and were mostly diagnosed with BD-I. Interestingly, among BD-I patients, 54.5% met the criteria for cPTSD, whereas among subjects affected by BD-II, only 16 out of 91 (17%) showed comorbid cPTSD. The duration of untreated illness is significantly shorter in the cPTSD group, suggesting that individuals with cPTSD sought treatment earlier.

A family history of psychiatric disorder was prevalent in individuals with cPTSD who showed a higher rate of various clinical features, including a greater number of total psychiatric episodes, aggressive behaviors, psychotic symptoms, and mixed features than patients without cPTSD. Also, cPTSD-positive patients showed more seasonality than cPTSD-negative patients, with a higher prevalence of lifetime abuse, antidepressant-induced mania as well as suicide attempts, indicating a potentially more complex clinical profile in this subgroup of BD patients.

**Table 4:** Description of enrolled BD patients with and without comorbid cPTSD, classified based on ITQ score, and results from the statistical comparisons between groups using t-test or chi-square as appropriate

Variables	cPTSD				*p*
	No *N* = 190		Yes *N* = 154		
Age, M (± SD)	48.7	± 13.48	44.65	± 14.21	**0.008**
Age at onset, M (± SD)	27.0	± 10.29	24.15	± 7.93	**0.004**
duration of untreated illness, M (± SD)	7.03	± 10.98	4.36	± 9.25	.**015**
number of depressive episodes, M (± SD)	5.71	± 6.84	5.94	± 4.56	0.71
number of manic episodes, M (± SD)	3.94	± 5.60	4.12	± 3.12	0.71
number of hypomanic episodes, M (± SD)	2.50	± 2.12	2.84	± 3.27	0.27
numbers of total affective episodes, M (± SD)	10.45	± 10.85	12.56	± 8.40	**0.043**
Alda Scale, M (± SD)	5.07	± 2.83	4.07	2.40	**< 0.001**
Female, N (yes%)	108	56.8%	69	44.8%	**0.026**
Graduation, N (yes%)	127	66.8%	119	77.3%	**0.033**
Marital status, N (yes%)	93	48.9%	93	60.4%	**0.034**
Employed, N (yes%)	96	50.5%	75	48.7%	0.74
Diagnosis of bipolar disorder I, N (yes%)	115	60.5%	138	89.6%	**< 0.001**
Diagnosis of bipolar disorder II, N (yes%)	75	39.5%	16	19.4%	**< 0.001**
Family history of psychiatric disorder, N (yes%)	91	47.9%	99	64.3%	**0.002**
Seasonality, N (yes%)	78	41.9%	93	60.8%	**< 0.001**
Aggressive behaviors, N (yes%)	83	43.7%	111	72.1%	**< 0.001**
Mixed features, N (yes%)	71	37.4%	122	79.2%	**< 0.001**
Lifetime abuse, N (yes%)	45	23.7%	79	51.3%	**< 0.001**
Psychotic symptoms, N (yes%)	14	7.5%	130	84.4%	**< 0.001**
Antidepressant mania, N (yes%)	20	10.5%	65	42.2%	**< 0.001**
Suicide attempts, N (yes%)	28	14.7%	110	71.4%	**< 0.001**

### BD patients with comorbid cPTSD show a worse response to pharmacological treatment

As shown in Table 5, we detected differences in the distribution of pharmacological therapy between groups. The p-value is < 0.001 and it suggests a significant difference in therapeutic approaches (polytherapy vs. monotherapy). A higher percentage of patients with cPTSD were on polytherapy, and a lower percentage were treated only with lithium compared to those without cPTSD.

To determine whether there was a different response to therapy between cPTSD-positive and cPTSD-negative BD patients we analyzed the data obtained from the Alda scale, and we detected a significant difference in the mean Alda score between groups (presence/absence of cPTSD). Specifically, BD patients with comorbid cPTSD show a lower level of mean Alda score compared to those without it.

Furthermore, as shown in Table 6, the results of the logistic regression model indicate that higher Alda scores (thus better treatment response) are associated with a decrease in the log odds of having cPTSD. Indeed, the intercept results show that for a one-unit increase in the Alda score, the odds of having cPTSD are 1.554 times higher. Conversely, BD patients with lower Alda scores (indicating poorer treatment response) are more likely to have comorbid cPTSD. The results of the estimate show that for a one-unit increase in the Alda score, the odds of having cPTSD decrease by a factor of 0.867. Overall, these findings suggest significant associations between therapeutic approaches, Alda score, and the presence of cPTSD in BP patients.

**Table 5:** Results of the analysis of the distribution of enrolled BD patients into different therapeutic approaches as well as of the Alda score between BD-patients with and without cPTSD

	cPTSD				*p*
	No *N* = 190		Yes *N* = 154		
Polytherapy, N (yes%)	146	76.8%	131	85.1%	**< 0.001**
Lithium, N (yes%)	44	23.2%	23	14.9%	**< 0.001**
Alda score, M (± SD)	5.07	± 2.83	4.07	± 2.40	**< 0.001**

**Table 6:** Results of the logistic regression analysis

		95% Confidence Interval						95% Confidence Interval	
Predictor	Estimate	Lower	Upper	SE	Z	p	Odds ratio	Lower	Upper
Intercept	0.441	0.0109	0.8702	0.2192	2.01	0.044	1.554	1.011	2.387
alda_tot	-0.142	-0.2240	-0.0603	0.0418	-3.40	< 0.001	0.867	0.799	0.941

Note. Estimates represent the log odds of “cPtsd = 1” vs. “cPtsd = 0”

## Discussion

The current study intended to assess the impact of cPTSD on the response to mood stabilizers (single drug or in combination) in an adult outpatient population with BD. It was reported that patients with BD are more likely than the general population to have suffered traumatic events, especially during childhood and adolescence, and that these experiences when extended over time, may be predictive for the development of cPTSD, representing a defined and serious risk factor (Tian et al. 2022). Moreover, the main differences between PTSD and cPTSD lie in trauma duration and symptoms. While PTSD can occur a single acute events like car accidents, cPTSD results from prolonged trauma. Both disorders involve stress responses like flashbacks and hypervigilance, but cPTSD includes persistent challenges in emotion regulation, identity, and relationships (Wang et al. 2024). These latter three domains characteristic of cPTSD can play a fundamental role in exacerbating the affective symptomatology of bipolar disorder and explain much of the results reported in Table 4. Further, there is consensus in the literature that a history of repeated trauma is common in individuals suffering from BD and can have a very negative impact on the course of the disease (Kira 2010; Shevlin et al. 2008). In line with this evidence, our cohort of BD patients with comorbid cPTSD exhibited more severe symptomatology, such as an earlier age at the onset of BD, a higher frequency of affective episodes, aggressive behavior, mixed features, psychotic symptoms, and a greater number of suicide attempts. These results are consistent with our recent study where we demonstrated how cPTSD predicts suicidal ideation in patients with BD. Not only that, but they also confirm all the unfavorable prognostic characteristics of BD, which could undermine the treatment response (Iazzolino et al. 2024). Of note approximately 45% of our sample of BD patients were classified as cPTSD-positive according to the ITQ also found a higher prevalence of cPTSD in patients diagnosed with BD-I compared to BD-II. This result supports our hypothesis, and of great importance is the difference observed in the emergence of suicidal ideation, which is likely due to the constant re-experienced of trauma faced, as cPTSD by definition refers to the exposure to a stressor typically of an extreme or prolonged nature and from which escape is difficult or impossible. Additionally, there is the presence of psychotic symptoms commonly emerge. This data could be explained by the fact that patients with cPTSD exhibit a greater symptom burden, particularly dissociative symptoms, which could predispose to the development of psychotic symptomatology (Steardo et al. 2021). Of great importance is the difference that emerges in aggressive behavior, as well as a higher incidence of mixed symptomatology, which could be due to disturbances in self-consciousness leading to increased emotional lability. Aggressive behavior may stem from the difficulty individuals with cPTSD face in regulating their emotions or from the frustration of frequently experiencing stressful situations, resulting in a poor quality of life. Furthermore, some factors would suggest a real vulnerability of bipolar patients with certain innate clinical characteristics predisposing to the development of cPTSD, and this could be hypothesized considering the differences observed between the groups. In the cPTSD group, there were early onset age, seasonality, and a higher number of affective decompensations.

All the above suggests that a history of multiple trauma precipitating cPTSD could play a relevant role in the psychopathology of BD, although the cellular and molecular mechanisms by which this comorbidity may influence the response to treatment remain to be elucidated.

The whole sample showed a moderate response to treatment on average (4.63 ± 2.69 *n* = 344). However, when the sample was further divided into two subgroups according to the presence/absence of cPTSD, the diagnosis of cPTSD was found to affect the response to treatment (5.07 for the sample without cPTSD; 4.07 for the sample with cPTSD, *p* < 0.001). These findings are consistent with previous results showing that childhood trauma per se can blunt treatment (Etain et al. 2017; Cakir et al. 2016; Cascino et al. 2021; Benarous et al. 2017; Perugi et al. 2018) and this may result in a greater likelihood of inadequate response to pharmacotherapy (Etain et al. 2017). Moreover, cPTSD as a comorbidity may also influence the outcome of the combined treatment with quetiapine and lithium in BD patients (Russell et al. 2023).

This study shows that the concomitant use of multiple mood stabilizers is common in this population, especially in patients with previous poor responses to treatment (Baek et al. 2014), and how this could not change a greater probability of weaker efficacy of therapy (Lee et al. 2020). Consistent with this, our patients with comorbid cPTSD tended to have a limited response to treatment with a single first-line agent (lithium), thus necessitating a combination, which still failed to modify the likelihood of a poor treatment outcome. Although, this is the first study to investigate the response to mood stabilizers in a group of patients with cPTSD compared to those who have experienced traumatic events and present PTSD. The difference in response between the two groups, with a lower score on the Ada scale in the cPTSD group, may also be due to the prolonged nature of the trauma and often the non-recognition of this diagnostic category because it is underestimated and poorly diagnosed.

The evidence that combined treatments with multiple medications were equally ineffective in bipolar patients comorbid with cPTSD is not without relevance considering the greater incidence of affective episodes as well as the more rapid cyclicity and higher frequency of mixed states. Above all, the significantly higher risk of suicide in these patients than cPTSD-negative patients must be considered, which we would counteract with less effective tools.

The findings of the present study should be read considering the following limitations. First, the cross-sectional design and the relatively small sample size may have limited the generalizability of the results. Patients were studied in different phases of illness and different settings. Despite this, the study mainly relied on the analysis of lifetime variables that were not affected by clinical severity at the time of assessment. Furthermore, an in-depth analysis of the cumulative trauma experienced by BD patients and the differential impact of the different types of trauma should be conducted. Indeed, Maercker and colleagues found significant differences depending on the type of trauma experienced by individuals who would develop PTSD (such as kidnapping and rape) compared to those who would develop cPTSD (childhood maltreatment and sexual and domestic violence) (Maercker et al. 2018). This will be the topic of our future research. A future study should compare different groups of cPTSD, PTSD, and BD without lifetime trauma history. Future prospective studies are required to clarify the association between BD and cPTSD and possibly identify specific risk factors and clinical phenotypes according to the BD subtype. Nonetheless, the major strengths of this study are that the focus of the study was very specific, as we analyzed the comorbidity of BD with cPTSD in a real-world setting, using broad inclusion criteria which contributed to the representativeness of the sample of the whole BD population. Furthermore, the present study confirmed specific clinical correlates of a history of trauma in BD, providing results about treatment response assessed with an instrument with high validity and interrater reliability. The results of the present study suggest that the comorbidity of cPTSD with BD worsens the course of both and reduces the therapeutic efficacy of mood stabilizers, administered alone or in combination, and stimulates further research studies to explore the neurobiological mechanisms responsible for the reduced response to pharmacological treatment. This could expand our knowledge and enable us to develop more tailored and effective treatments that are suited to the characteristics of patients.

The possible occurrence of cPTSD in subjects suffering from BD should be carefully researched starting from the onset of the disease since this simultaneous association leads to greater clinical severity and should prompt close monitoring of response to treatments because they prove to be less satisfactory in this condition. Moreover, an add-on treatment specifically targeted to trauma could result in a better outcome and response in this population of BD. Several interventions that have been shown to be effective in trauma therapy for patients with bipolar disorder, including various techniques such as eye movement desensitization and reprocessing, dialectical behavior therapy, and mindfulness-based cognitive treatment (Hett et al. 2022). However, as highlighted in a recent meta-analysis by Karatias and colleagues, trauma-focused interventions, which are more commonly used in PTSD, yield poorer responses in cPTSD. Hence, there is also a need to develop a tailored intervention for this syndrome (Karatzias et al. 2019b). The implementation of personalized interventions is required to optimize the clinical management of BD patients with c-PTSD.

## Data Availability

No datasets were generated or analysed during the current study.

## Abbreviations

AP: Antipsychotic
BD: Bipolar Disorder
cPTSD: Complex Posttraumatic Stress Disorder
DSO: Disturbances in Self-Organization
DVP: Valproic Acid
ITQ: International Trauma Questionnaire
LI: Lithium
MS: Mood Stabilizer
PTSD: Posttraumatic Stress Disorder
